# Technologies to monitor the health of loaded skin tissues

**DOI:** 10.1186/s12938-018-0470-z

**Published:** 2018-04-12

**Authors:** Dan L. Bader, Peter R. Worsley

**Affiliations:** Skin Health Group, Faculty of Health Sciences, University of Southampton, Southampton General Hospital, Tremona Road, Southampton, SO16 6YD UK

## Abstract

There are many situations where the skin and underlying soft tissues are compromised by mechanical loading in the form or pressure, or pressure in combination with shear. If sustained, this can lead to damage in the tissues particularly adjacent to bony prominences, resulting in chronic wounds. An array of bioengineering technologies have been adopted to assess the integrity of loaded soft tissues. This paper aims to review these approaches for the quantification, simulation and early detection of mechanically-induced skin damage. The review considers different measurements at the interface between the skin and support surface/medical device, involving pressure, shear, friction and the local microclimate. The potential of the techniques to monitor the physiological response of the skin to these external stimuli including biophysical measurement devices and sampling of biofluids are critically analysed. In addition, it includes an analysis of medical imaging technologies and computational modelling to provide a means by which tissue deformation can be quantified and thresholds for tissue damage defined. Bioengineering measurement and imaging technologies have provided an insight into the temporal status of loaded skin. Despite the advances in technology, to date, the translation to clinical tools which are robust and cost effective has been limited. There is a need to adapt existing technologies and simulation platforms to enable patients, carers and clinicians to employ appropriate intervention strategies to minimise soft tissue damage.

## Background

### The structure and function of skin

The skin represents the largest organ of the body, with its structure being divided into three separate layers; the epidermis, the dermis and subcutaneous tissue. The former outermost layer is approximately 75–150 μm thick, although it is considerably thicker in the palms of the hands and plantar aspects of the feet. The epidermis is divided into five strata, the deepest of which is the region in which the keratinocytes, the main epidermal cells, proliferate and slowly progress through the strata. The most superficial layer, the stratum corneum, consists of 15–20 layers of dead anucleated cells, termed corneocytes. The other cell types include melanocytes, producing the colour pigment, melanin, Langerhan cells responsible for immune response and Merkell cells that provide tactile sensation.

The integrity of the epidermal–dermal junction, an undulating structure, is critical for the normal transport and communication of biomolecules between the epidermis and the underlying dermis [1]. The human dermis contains many structural features including blood and lymphatic vessels, nerve endings and skin appendages, such as hair follicles, sebaceous glands and sweat glands. The fibroblasts produce extracellular matrix components, collagen, elastin and hydrophilic proteoglycans, which vary within the reticular and papillary dermal layers. The subcutaneous tissue, or hypodermis, is a fibro-fatty layer loosely connected to the dermis, which varies with anatomical site, age, gender, race, endocrine and nutritional status of the individual. Subjacent to this layer can be a muscle layer, which overlies either bony prominences or internal tissues and organs.

Functionally, the highly organised skin is designed to permit gas/fluid transport across its surface and, critically, maintain the internal body homeostasis, via the sweat glands and blood vessels. Other functions include protection of underling tissues and organs, excretion, immunity and synthesis of vitamin D [2]. These functional roles can be compromised by the external environment where the skin is exposed to a range of insults, which may be mechanical, physical, biological and chemical in nature. As an example, when the skin is exposed to high mechanical loads applied over a short time period (< 10 s), trauma can occur. By contrast, there are many situations in which the skin can be exposed to sustained mechanical loads, for example in individuals who are relatively immobile and bedridden or function in chairs for much of their waking day. Prolonged and cyclic loading is also experienced during activities of daily living (ADL) such as standing and walking.

### Pressure ulcers and diabetic foot ulcers

Prolonged loading can lead to damage of skin and subcutaneous tissues and result in conditions termed either pressure ulcers (PUs) or diabetic foot ulcers (DFUs). PUs may be defined as a localized injury to skin and/or underlying tissue, usually over a bony prominence, as a result of pressure, or pressure in combination with shear [3]. PUs are generally categorised in terms of the extent of the associated soft tissue damage. Thus PUs confined to the epidermal tissues are referred to as grade (or stage) I ulcers. Grade II ulcers affect deeper dermal tissue, although with effective management, generally heal successfully. By contrast, damage affecting subcutaneous tissues is classified as grade III and IV, which may account for approximately 30% of the total reported [4]. Another type of PU, termed deep tissue injury (DTI), is a pressure-related injury to subcutaneous tissues under intact skin. DTIs are typically seen in regions where tissue damage occurs adjacent to bony prominences i.e. the ischial tuberosity and the wound progresses upwards towards the skin.

Diabetic foot ulcer is an outcome of a complex array of various risk factors such as peripheral neuropathy, peripheral vascular disease, foot deformities, arterial insufficiency, trauma and impaired resistance to infection [5]. The lifetime risk of a diabetic for developing a foot ulcer can be as high as 25% [6], with DFUs accounting for more hospital admissions than any other long-term complications of diabetes [7]. As a result, the rate of lower limb amputations is 6 times higher in diabetic patients compared with non-diabetics [8].

**Fig. 1:**
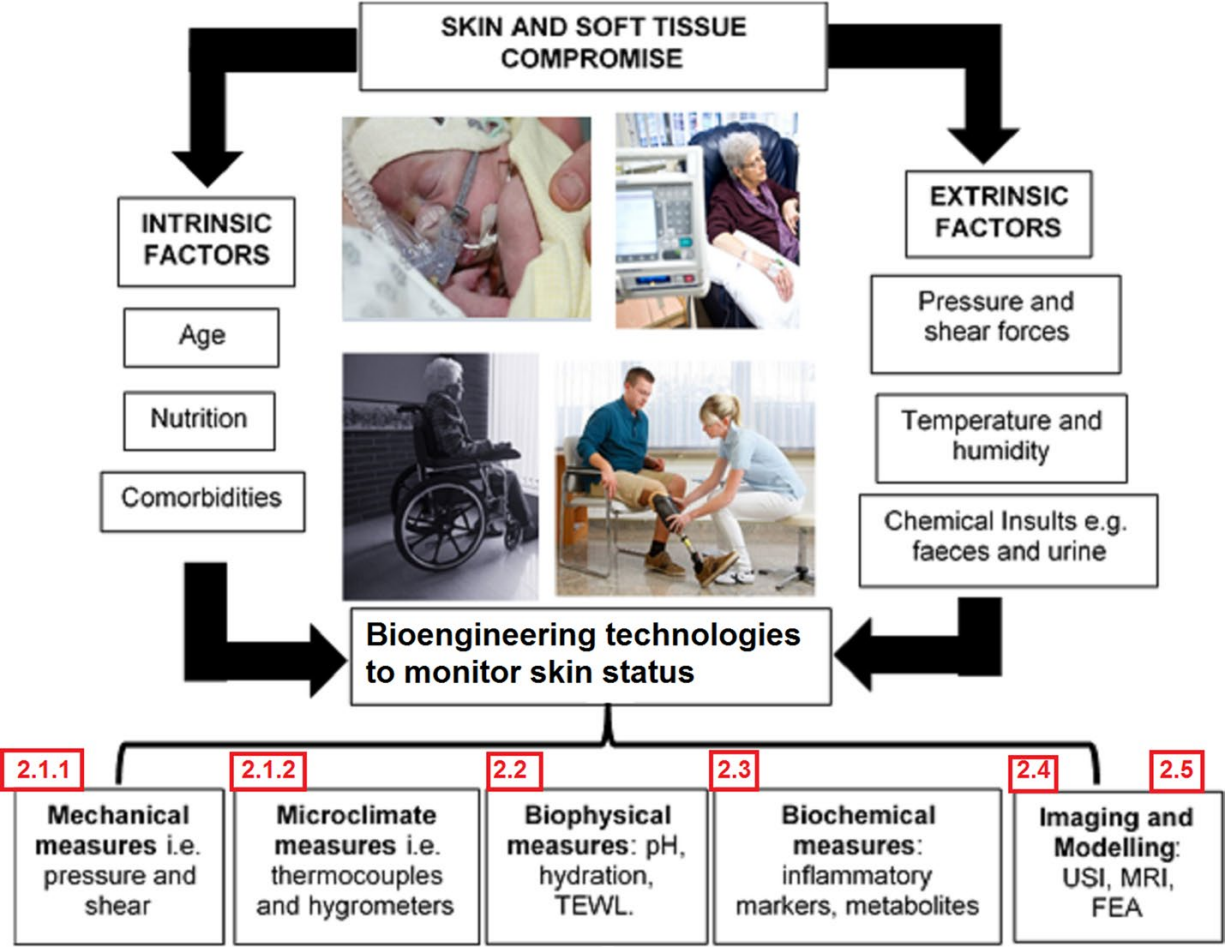
Schematic of the intrinsic and extrinsic factors that predispose individuals to skin damage and the bioengineering measurement techniques which can monitor their tissue status. The numbers relate to the sections describing the different technologies

These chronic wounds have been traditionally associated with the elderly, particularly those who have limited mobility. However, PUs affect a wider age range including neonates nursed in incubators [9], paediatrics and adults in intensive-care units (ICUs) [10] and the spinal cord injured [11]. Despite the increased attention within health services, PU and DFU incidence rates remain unacceptably high with corresponding costs of treating all chronic wounds estimated at £5 billion per annum in the UK [12]. In order to gain further insight into their prevention, bioengineering researchers have identified mechanisms by which skin and soft tissues are damaged during prolonged mechanical loading using in vitro and in vivo test methodologies. This knowledge was acquired using an array of techniques including medical imaging, physical sensors, biosensors and computational modelling to examine tissues in healthy and diseased/damaged conditions (Fig. 1). This review aims to critically appraise approaches for the quantification and simulation of mechanical conditions at the loaded skin surface and provide an evaluation of techniques which can monitor the risk of skin damage.

### Pathogenesis of pressure and diabetic foot ulcers

The aetiopathogenesis of PUs has long been considered to involve the obstruction of blood vessels within loaded soft tissues leading to pressure-induced ischemia. This mechanism will result in a limited delivery of vital nutrients, such as oxygen, to the cell niche. The resulting cell death would impede any remodelling processes and lead to the accumulation of soft tissue breakdown. However, compelling research utilising bioengineering technologies has revealed that PUs can result from other mechanisms namely:

- Impaired interstitial and lymphatic flow—this will result in an accumulation of toxic intercellular waste products, which are both damaging to the cells and can influence the local cellular environment e.g. reduced levels of local pH [13–15].
- Ischaemia–reperfusion injury associated with load removal—this results in the reperfusion of blood and transport of other nutrients, which may result in an over production and release of oxygen-derived free radicals, also termed reactive oxygen species (ROSs), which have been implicated in soft tissue damage [16, 17].
- Cell deformation—this triggers several effects, which may be involved in early damage, such as local membrane stresses leading to buckling and rupture. This loss of membrane integrity will alter transport of biomolecules and ions, cause volume changes and modifications of cytoskeletal organisation, affecting viability and remodelling capacity [18–20].

There are similarities but also some marked differences between the aetiology of DFUs and that of pressure ulcers. Fundamentally, the presence of pressure and shear, applied repetitively to tissue sites with a reduced tolerance to mechanical loading is likely to lead to damage to soft tissue areas adjacent to bony prominences, such as the sacrum and metatarsal heads. Such a situation can be exacerbated in the presence of elevated temperatures and moisture levels commonly encountered within the shoe. At particular risk are those individuals with associated soft tissue and bony deformity conditions, such as Charcot’s foot. In addition, comorbidities resulting from diabetes can lower the tolerance to skin and soft tissues loading, namely peripheral vascular disease, peripheral neuropathy or both [21]. A comparison of features associated with the two chronic wounds is provided in Table 1.

**Table 1:** Causation and management of pressure ulcers and diabetic foot ulcers

	Pressure ulcer	Diabetic foot ulcer
Prime responsibility	Nurse	Podiatrist
Causation—mechanical	Pressure, shear and friction Prolonged loading	Pressure, shear and friction High rate loading applied in a repetitive manner Ill-fitting shoes
Causation—microclimate	Temperature, humidity, incontinence	Temperature, humidity
Intrinsic factors	Immobility Insensitive	Structural deformity e.g. Rheumatoid, Charcot’s foot Neuropathic, Peripheral arterial disease
Tissue susceptibility	Reduction in stiffness Tissue atrophy	Increase in stiffness Tissue migration
Management	Immersion, pressure redistribution, alternating pressure	Pressure redistribution, total casts

## A bioengineering approach measurement and simulation

From a bioengineering perspective there are a number of technologies which can be used to monitor the status of loaded soft tissues. These include:

1. “Monitoring of the interface” section (Fig. 2).

**Fig. 2:**
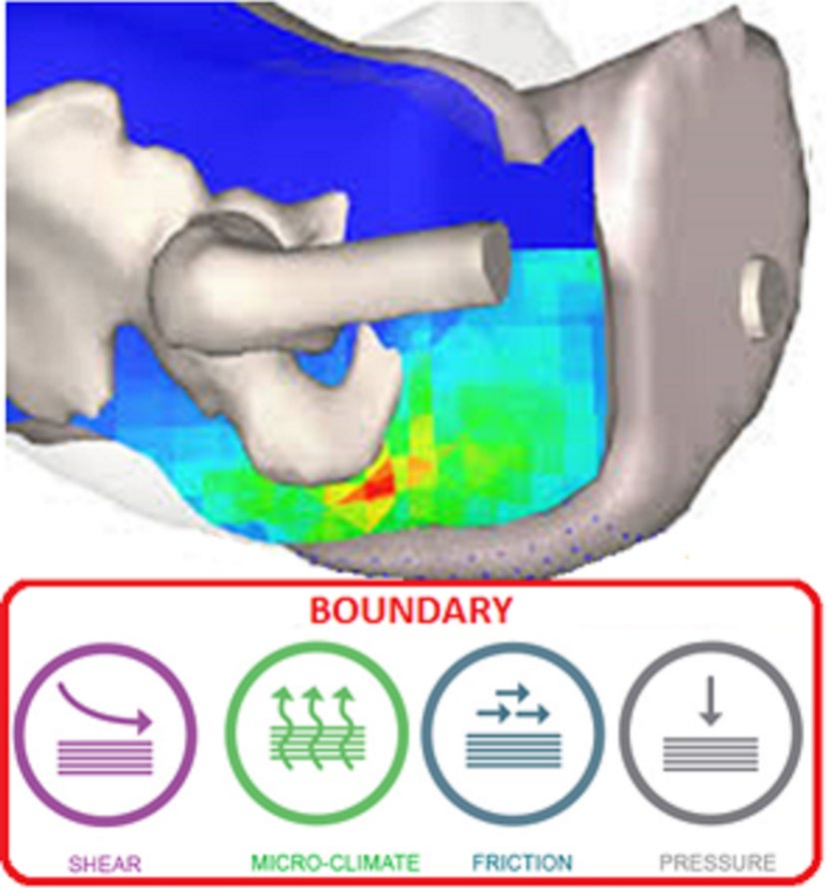
Factors influencing tissue health at the boundary between the support surface and the skin surface
2. “Biophysical skin sensing” section.
3. “Biomarkers indicative of early skin damage” section.
4. “Medical imaging of mechanically loaded tissues” section.
5. “Computational modelling” section.

### Monitoring of the interface

**Fig. 3:**
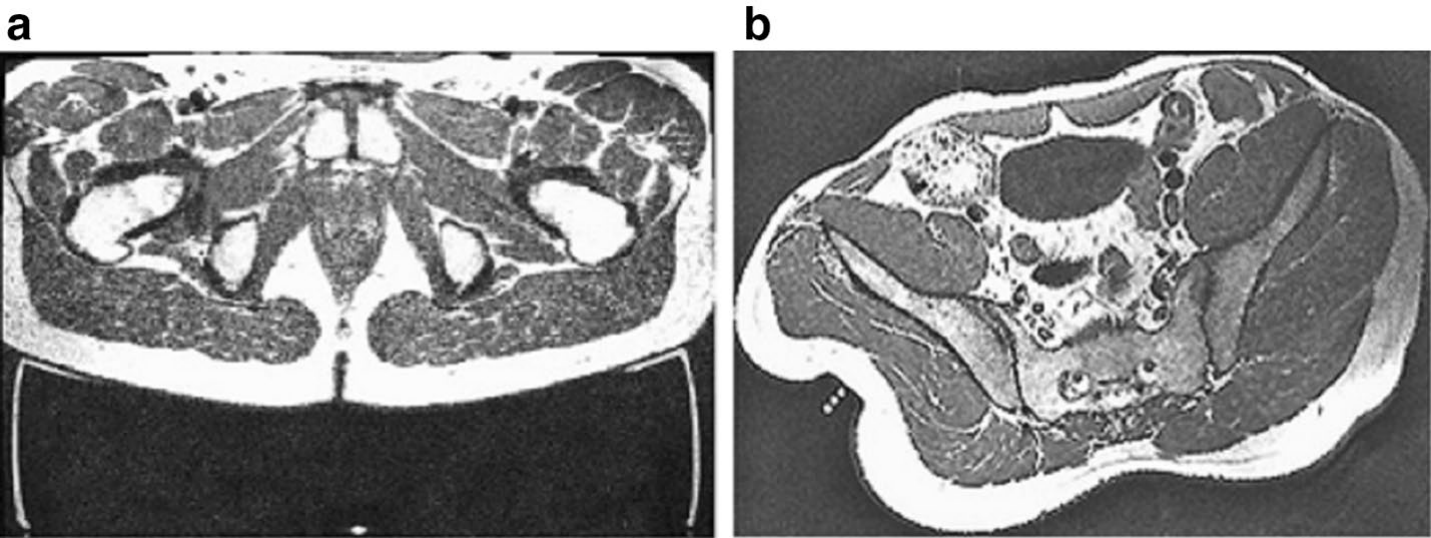
**a** MRI of seated buttocks and **b** gluteal muscle subjected to maximum indenter displacement with highlighted markers [146]

There is a critical relationship between the magnitudes of pressure and time which can result in skin damage [22]. Early research established an integral of pressure and time above which damage would occur [23]. More recently, this model has been adapted to match a sigmoidal form, which accounts for tissue damage resulting from high tissue deformations occurring after a short period [24]. This relationship inevitably depends on the tolerance level of the individual which is, itself, influenced by co-morbidities and nutritional status [25]. The risk of skin and soft tissue damage will also be affected by the way in which load is transferred across the skin surface. Indeed, if the load is non-uniform or localized in nature tissue damage is more likely than if the load is distributed uniformly. Specifically, these non-uniform loads cause internal shear stresses in the underlying tissues, which act to distort tissues, pinch and occlude capillaries crossing tissue planes, reduce blood and lymph flow and cause physical disruption of tissues [26]. For example, imaging demonstrates the difference in soft tissue deformation at the seated buttocks (Fig. 3a) compared with that of the gluteal muscle that has been indented with a small diameter device (Fig. 3b). In the former case, where the force is applied across the whole gluteal area the deformation in the underlying skin, fat and muscle tissue is relatively evenly distributed. However, in the case with the indenter, high deformations can be observed directly under the load and internal shear forces in the tissues adjacent (Fig. 3b). It is therefore critical to understand both the spatial and temporal nature of the pressures applied to the body.

#### Interface pressure mapping

Sensor arrays have been developed to estimate the distribution of pressures for use in both research and clinical settings. Pressure mapping systems are often used at discrete time points offering a “snap shot” of the interface conditions. This provides a limited perspective of the long-term performance of support surfaces and the effects of sub-optimal postures, e.g. slumped sitting, which are commonly adopted over time [27]. Recently mapping systems have been adapted to record data over prolonged periods [28]. These systems can provide visual feedback for repositioning patients and indicators for patients, carers and healthcare professionals regarding the exposure to prolonged loads on vulnerable skin sites [29, 30]. However, more research is required to establish algorithms, which correlates the pressures monitored over prolonged periods with changes to the physiological response of the underlying skin and soft tissues. In addition, standards for spatial resolution, sampling frequency, accuracy, sensitivity and calibration need to be established [31]. Pressure mapping also provides real-time visual feedback of peak pressure values, providing further evidence to complement decision making when considering PU prevention [32]. The transferable nature of the sensors has enabled clinicians to assess the effects of posture and mobility in various bed and wheelchair or leisure chair environments [33].

The results from pressure mapping studies have shown that the recorded values depend on the individual, their posture and the type of support surface [34–36]. For example, when lying supine on a typical hospital bed with a viscoelastic foam mattress, pressures over the sacrum rarely exceed 50 mmHg [34]. However, on a much stiffer surface, such as a spine board, supine pressures can exceed 150 mmHg [37]. In sitting postures, where contact areas are restricted to the ischial tuberosities (ITs) and buttocks, the sacrum and upper thighs, there is a corresponding elevation in interface pressures [38]. In a separate example involving a ventilation mask attached firmly to the face, interface pressures can exceed 200 mmHg over vulnerable bony landmarks, such as the nasal bridge [39]. Plantar pressures under the foot are necessarily very high during both standing and gait activities with values of up to 1500 mmHg specifically under the metatarsal heads [40].

#### Shear and friction measurement

In contrast to pressure mapping, there is a dearth of studies monitoring shear forces at the individual-support surface interface. This is mainly due to the technical challenges inherent in developing compliant, thin and flexible sensors that can distinguish between signals associated with normal forces with those forces acting parallel to the skin surface. Recently research has exploited the use of 3D printing with elastomeric materials to create sensors, which are capable of simultaneous measurements of pressure and shear forces [41, 42]. These sensors have been developed for the specific application at the stump-socket interface of lower limb amputees, where peak shear forces during gait were reported to be approximately 27 kPa. The modification of these biaxial sensors for the measurement of the inherently lower shear forces predicted at the sacrum and ITs in the lying and sitting postures have yet to be described.

The shear force at the individual-support surface interface will be in part dependent on the friction between the two surfaces. The coefficient of friction of materials, commonly textiles, against skin is influenced by:

- the textile characteristics i.e. rougher textiles produce higher coefficients of friction.
- skin moisture content and surface—both increase the coefficient of friction where skin may be damp from perspiration or incontinence [43].

There have been several experimental studies to determine the coefficient of friction in typical support surfaces [43, 44]. In many cases, hospital mattresses incorporate polyurethane covers to enable safe cleaning and minimise the intrusion of liquids into the foam, gel or air inner. However, this covering material restricts vapour transfer through the skin interface resulting in elevated temperatures and the accumulation of body fluids such as sweat [45, 46].

#### Microclimate measurement

Studies have employed thermocouples, thermography and hygrometer devices to monitor the microclimate at a loaded skin interface [47]. They have revealed elevated temperature and humidity values in the plantar aspects of the foot [48], the residual amputee stump-socket interface of amputees [49] or at tissues where high forces are transmitted through foot orthoses [50]. These changes will inevitably reduce the skin tolerance to mechanical-induced damage [51–53]. For example, temperatures in excess of 35 °C have a detrimental effect on the stratum corneum by affecting its mechanical stiffness and strength [54]. Skin temperature also affects the local tissue physiology, with a 1 °C rise resulting in a 13% increase in the metabolic demand [47], providing additional risk to vulnerable soft tissues already compromised by local vascular and lymphatic occlusion. Increased skin moisture also contributes to maceration and skin breakdown by weakening the stratum corneum, [55, 56]. Conversely, an excessively dry skin is liable to damage by cracking [57]. Thus, achieving an optimal moisture level at the skin interface is critical for maintaining its barrier function.

#### Limitations of interface measurement technologies

In addition to the variability of interface measurements, the large data sets produced from pressure mapping systems are difficult to interpret. Indeed, the establishment of a robust pressure index appropriate in research and/or clinical settings is the subject of considerable debate [58]. For example, common interface pressure parameters include peak pressure, peak pressure gradient, peak pressure index, dispersion index, average pressures and symmetry index, the latter comparing values on two sides of the body, in addition to spatial parameters, such as contact area and centre of pressure [59, 60]. However, it is inevitable that no single parameter of pressure, shear or microclimate can provide universal index applicable to all subjects at risk of developing PUs or DFUs. In order to understand how interface conditions affect local skin physiology several studies have combined biomechanical, biophysical and biochemical measures to define the effects of prolonged mechanical loading [34–36, 39].

### Biophysical skin sensing

The effects of mechanical loading and/or altered microclimate on barrier function of the skin can be evaluated with a range of techniques involving transepidermal water loss (TEWL), pH, subepidermal moisture (SEM) [61], elasticity and colorimetry [62]. These studies generally reveal that sustained loading increases TEWL at various skin sites, suggesting sub-clinical damage of the stratum corneum [63, 64]. These increases will result in an increase in the dermal absorption of chemicals and other potentially toxic substances [65]. In addition, systems to measure SEM, elasticity and redness have been reported to detect changes between healthy skin and sites of pressure ulcers, although they were unable to determine the extent of the damage [61, 66, 67]. However, such biophysical measures are associated with a number of challenges. For example, there is variability in both intra- and inter-rater reliability [68] and regional differences in baseline values [69]. In addition, there is very little data regarding the sensitivity and specificity of the techniques to distinguish between mechanical, chemical or environmentally-induced skin damage. Thus although longitudinal studies are recommended, optimal measurement procedures and test protocols still need to be established if specific techniques are to be translated into clinical practice.

Monitoring the ischemic response in the dermal vasculature during and after mechanical loading typically involves physical sensors, sensitive to both direct and indirect measures of blood flow. These studies, often utilising transcutaneous gas tensions (TcPO_2_ and TcPCO_2_) measurements, have examined the response of able-bodied cohorts to periods of prolonged and intermittent pressures [35, 36, 70, 71], and sub-groups of patients known to be at risk of DFUs [72] and pressure ulcers i.e. spinal cord injured (SCI) subjects [73]. The results of these studies revealed ischemic responses, as reflected in a reduction in T_c_PO_2_ with an associated increase in T_c_PCO_2_, during postures known to create both pressure and shear forces at vulnerable sacral tissues, for example, when the head of bed angle has raised to 45° [36, 70]. Indeed, the increase in TcPCO_2_ is hypothesised to be a critical indicator of skin and soft tissue compromise [74]. Microcirculatory flow has been explored in a number of PU and DFU-related studies using laser Doppler (LD) technologies. As examples, it has been shown that microcirculatory flux differs in the feet of diabetic patients with and without neuropathy [75] and can distinguish between areas of undamaged skin with sites of PUs and DFUs [76–78]. In addition, LD flux measurements have revealed that the combination of pressure and shear decreased local tissue perfusion [79]. LD imaging has also provided a means to assess burn depth [80] and is sensitive to chemical irritation on skin sites [81]. However, the arbitrary units of flux derived from these measurements can not be directly related to physiological parameters and are strongly influenced by motion artefacts, ambient temperature changes and inter-operator variability.

### Biomarkers indicative of early skin damage

There are a number of biofluids, which can be collected directly at the skin surface or systemically in blood or urine, for which a number of biomarker concentrations can be analysed. These biomarkers can be targeted to represent inflammatory processes [C-reaction protein (CRP), cytokines and chemokines], local metabolic activity (metabolites) or the release of oxygen free radicals during reperfusion (purines). Previous studies on both healthy volunteers and individuals at risk of developing PUs, have demonstrated the potential of some biomarkers, for example, sweat lactate [82], the pro-inflammatory cytokine, IL-1α [83] and CRP levels in blood [84].

Seminal research has combined transcutaneous gas tension measurement with biomarker analyses from sweat to evaluate the effects of different loading regimens on able-bodied individuals [82]. The authors revealed a significant relationship between the reduction in TcPO_2_ and an elevation of sweat lactate a marker of anaerobic cellular respiration. In addition, the findings revealed that during localised skin loading in excess of 80 mmHg (10.7 kPa), there was considerable accumulation of TcPCO2. In addition, above a threshold of pressure and loading time, there was a distinct elevation of sweat lactate and urea. Recently, analysis has revealed that there is also a temporal change in the ratio between lactate and pyruvate concentrations in sweat sampled pre- and post-mechanical loading [85]. Concentrations of sweat biomarkers indicative of reperfusion injury, namely purines, have also been reported to be sensitive to periods of mechanically induced-ischemia [86]. Sophisticated chromatographic techniques have been more recently employed to measure both metabolites and purines in small concentrations, allowing for quantitative analysis of several analytes to be performed simultaneously [87]. Subsequently, the potential to interrogate the biochemical milieu of skin and soft tissues to provide an early indicator of potential damage has become an emerging area of interest.

Cytokines, which are derived from active keratinocytes in the epidermis may be collected from sebum at the skin surface using specially designed tapes. These commercially available tapes are applied to the skin for short periods (2 min) and the sebum is extracted in a solution of saline with additional non-ionic surfactant, Tween, using sonication. The extracts can be analyzed for human cytokines using commercial immunoassay test kits. The cytokine levels recovered from each tape extract are generally normalized to total protein (TP) levels. This mechanism has yielded a number of recent studies involving the response of skin tissues to prolonged loading via medical devices e.g. respiratory masks [39] and spine boards [88], as well as the combined effects of prescribed shear and pressure [83]. However, certain technical limitations remain before appropriate robust biosensors could be incorporated into routine screening protocols and used in conjunction with traditional risk assessment scales. These include limited sample volumes, low concentration levels, particularly for cytokines and the temporal profiles and interaction of the biomarkers. The advent of low cost highly sensitive portable point-of-care (PoC) testing systems based on printed electrochemical sensors could provide a means of clinical translation. Although biomarkers sampled from the skin surface provide a means to examine the status of both epidermal and dermal tissues, it provides little indication of compromise to the underlying subcutaneous and muscle tissues. Biomarkers of deep tissue injury, specifically concerning muscle cell damage, have been identified in blood where CRP levels were significantly raised in SCI subjects with PUs [89].

### Medical imaging of mechanically loaded tissues

The relationship between interface pressures and the resulting internal mechanical state involving the interstitial stresses/strains is necessarily complex in nature. It is dependent on the thickness of the tissue layers and the mechanical and anatomical characteristics of the tissue composite between the skin surface and the bone [90]. In order to quantify the distortion of skin and soft tissues, medical imaging modalities can provide quantitative, volumetric data of loaded tissues. The relative volume changes in skin, fat and muscle can provide an indication of how load is transferred through soft tissues and provide a basis to relate mechanical loading to pathophysiological events within deeper tissues. Imaging studies of mechanically compromised tissues have included both animal [91–93] and human models [26, 94–96]. These have utilised a number of modalities, each of which will be discussed separately.

#### MRI-based studies

During MRI scanning, protons (hydrogen atoms) in tissues containing water molecules create a signal that is processed to provide high contrast images of soft tissues. Using animal models, MRI has been used to reveal prolonged and intermittent loading can cause muscle oedema, inflammation and structural damage [20, 93, 97–100]. Accordingly, T2 MRI data has been established as a quantitative damage marker in musculoskeletal MRI. In addition, MRI has been used with both able-bodied and at risk sub-groups in clinical settings. These include the interface between a socket and residuum in amputees [101], supine postures on a spine board [94], the loaded plantar surface of the foot [102] and seated postures in healthy and spinal cord injured patients [90, 103]. These studies have revealed high levels of tissue deformation during commonly adopted postures e.g. sitting and lying. This deformation appears to be dependent on factors such as the characteristics of the support surface [104], the underlying anatomy [105] and the multidirectional translation of soft tissues [106].

#### CT-based studies

CT imaging offers a continuous scanning method to provide full volumetric, quantitative data. Several studies have used CT scans to image loaded soft tissues and provide accurate tissue geometry for computational models simulating pressure ulcer risk [90, 107–109]. The resulting high contrast images of bony anatomy distinguishes between trabecular and cortical bone, offering the potential to accurately assess deformity of foot structures which may predispose individuals to DFUs [110]. However, CT imaging is associated with radiation exposure and the image sequences are limited in differentiating between the individual soft tissue structures [111].

#### Other imaging-based studies

High frequency dermal ultrasound imaging (USI) has been used to investigate underlying tissue changes involving the presence of oedema in the deep sub-dermal and superficial dermal layers prior to skin breakdown [112]. USI is portable and can be incorporated into a 3D printed orthotic device to examine the functional behaviour of the foot during gait [113] and hence the performance of off-loading orthotic devices to prevent DFUs. US measurements have also shown promise for risk assessment to guide clinicians in appropriate interventions to prevent DTI, with measures corresponding to MR image data [114]. The technique has also been shown to be reliable when assessments of tissue composition are made offline [115]. However, it has been recently reported that although real-time interpretation of images related to muscle and fat are highly reliable, this is not the case for skin and bone morphology [116].

USI and MRI scanning techniques can also be used in conjunction with mechanical systems which displace the skin and soft tissues with a prescribed shear wave. The resulting deformation patterns, termed elastograms, enable quantitative values of shear modulus to be estimated, which depict local tissue elasticity or stiffness [117, 118]. Subsequently, magnetic resonance elastography (MRE) has been used to evaluate material property changes in foot fat pads of individuals with and without diabetes [119]. MRE was also used in an animal model to demonstrate changes in local tissue shear storage modulus of muscle exposed to damage-inducing indentation [120]. Experimental studies have also proposed ultrasound elastography (USE) as a promising technique to detect PUs at an early stage [121]. However, there are many practical issues to be resolved before technologies can be used routinely in a clinical setting to assess skin damage.

In order to determine the effects of mechanical loading on impaired lymphatic flow (“Pathogenesis of pressure and diabetic foot ulcers” section), seminal experimental studies have been conducted using lymphoscintigraphy with a canine model [122, 123]. These authors reported that both impaired lymphatic clearance occurred at an uniaxial pressure above 60 mmHg (8 kPa) and subsequent recovery of lymphatic clearance was highly dependent on the magnitude of the post-occlusive pressure [123]. Adopting a similar approach with radioisotopes is contraindicated in human volunteers due the inevitable risks associated with radiation exposure. However, recent research adopting the less invasive approach of near infra-red (NIR) fluorescence imaging, has revealed distinct changes in both the local interstitium and surrounding superficial lymphatic vessels following a period of loading [124, 125]. Further research is required to establish critical thresholds of both pressure and shear, which reliably occlude lymphatic vessels and damage associated lymph valves.

### Computational modelling

**Fig. 4:**
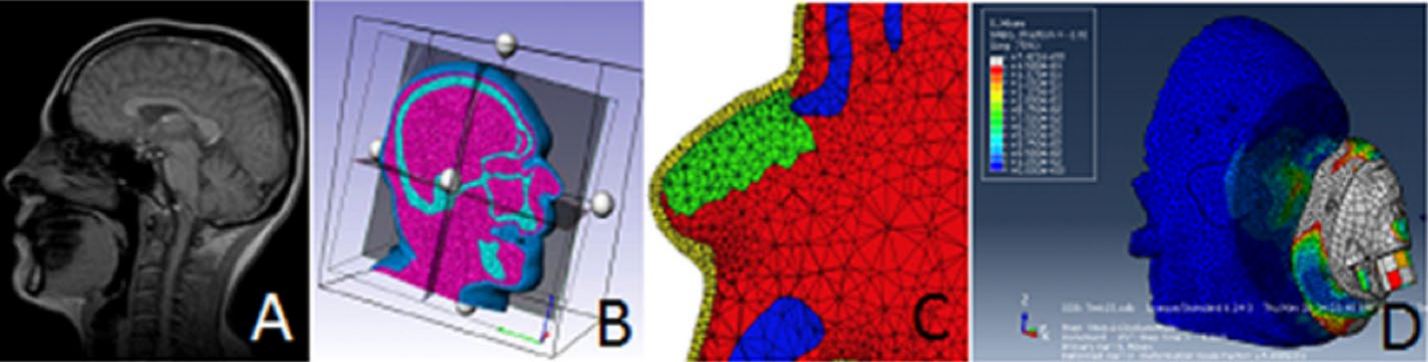
Conversion of CT stacked images (**A**) into a mesh containing tetrahedral elements (**B**). **C** Material properties are assigned to the model including skin (yellow), bone (blue), cartilage (green) and muscle (red). **D** Von Mises stress on the skin and medical device (respiratory mask)

The input of experimentally derived data including boundary conditions (“Monitoring of the interface” section), subject-specific biomechanical properties and tissue geometry (“Medical imaging of mechanically loaded tissues” section) have been incorporated into finite element (FE) models to simulate various clinical situations. This computational approach provides a means to estimate the internal mechanical conditions within loaded soft tissues. The FE approach has enabled pre-clinical analysis of medical device-skin interactions, providing a platform for sensitivity analyses to optimise designs of interfaces and effective offloading regimes [126]. Bony and soft tissue geometry can be defined using the volumetric data from imaging technologies (Fig. 4A), with proprietary software designed to translate stacked images into a mesh containing tetrahedral or hexahedral elements, using linear or quadratic shape functions (Fig. 4B). Although muscle, tendon, fat pads and ligament borders are visible using MRI, theses tissues are often modelled as one homogenous material to allow for convergence of tissue geometry. This approach, although computational efficient, creates an assumption that mechanical behaviour is uniform across these different structures. Indeed adding geometric detail (Fig. 4C) on a subject-specific basis provides the basis to estimate regional mechanical behaviour [127]. As an example, a two dimensional FE model of a transverse section of a transfemoral amputee has been developed [128]. The authors reported that the predicted stress magnitude in the residuum increased by 60% when different material properties were assigned to the muscles, inter-muscular tissues and the fascia. In addition, when the muscles were permitted to slide against other soft tissues, the peak stresses reduced by approximately 20%.

The successful implementation of FE analysis is highly dependent on the quality of material data characterising the behaviour of human tissues. Material parameters are often selected based on animal models, assuming that parametric values approximate those in human tissues [129]. For skeletal muscles data from in vivo animal experiments have been used [130, 131], while skin properties have been derived from both animal [132] and human data [133]. By contrast, there is very little data describing adipose tissue [134]. These studies confirm that the response of soft tissues to loading can be characterised as non-linear and time dependent. In order to account for the former, hyperplastic models have been developed, which will yield a strain energy density (SED) function. The SED function contains constitutive parameters, which represent material constants, as derived from experimental data [133]. FE studies have cited material characterization reports to provide these values, although the range of investigations cited has resulted in a large range of SED parameters being employed [135]. The incorporation of both non-linearity and time-dependency into a continuum material model represents a highly complex mathematical problem and, as such, has led to a range of approaches. For example, Portnoy et al. [95] used a neo-Hookean material model to represent the hyperelastic response of muscle tissues. Following the calculation of the strain energy density from the FE model, viscoelasticity has been derived from a Prony expansion.

Despite the challenges of converting experimental data to FE models, the computational approach offers significant insight into the mechanical behaviour of skin and soft tissues under varying loading regimes. The models have demonstrated how tissues are highly deformed under prolonged loading, resulting in soft tissue strains regularly exceeding 50% [106, 136], which are comparable to those estimated in MRI images. It is not known if these shear strain values correspond damage to the skin and underlying soft tissues in humans. However, animal testing has revealed that strains above a threshold could cause direct deformation damage of skeletal muscle [137] and strain may provide greater insight into the risk of deep tissue injury compared to pressure mapping measures at the skin interface [138]. To date, researchers have used FE models to examine the effects of support surface design [139–141] and microclimate at the interface [142–144], prophylactic dressings [145], insole performance for diabetic patients [146] and medical devices attached to skin tissues [143] (Fig. 4d). Authors have also used FEA as a platform to perform sensitivity analyses on parameters which mimic pathophysiological changes associated with chronic disease, including scar tissue [144], bone shape adaptation and muscle atrophy [139]. Although the clinical translation of these simulations has been limited to date due to the complexity within the models, several authors have attempted to simplify the process to provide real-time feedback during clinical situations. For example, Luboz and colleagues created a simplified FE model which provides personalised modelling for real-time pressure ulcer prevention in the sitting posture [145].

However, the interpretation of these models should be made in the light of the assumptions employed for both the geometry and material properties of the simulation. The clinical value will remain limited until stringent validation has been completed.

## Conclusions

Based on the technologies presented in this review, it is evident that there is a considerable armory of bioengineering techniques available to assess the effects of mechanical loading on the integrity of skin tissues. They are based on measurements at the skin-device interface, the physiological and biological response at the skin, as well as the imaging and modelling of the internal tissue status. The combination of these distinct technologies has provided the basis to predict the conditions which can lead to skin and soft tissue damage in a range of clinical situations e.g. pressure ulcers in sitting or diabetic foot ulcers during gait. Future research should focus on the translation of these technologies to provide robust, cost effective means by which individuals can be monitored over prolonged periods and targeted interventions delivered to those who are at high risk of tissue damage. In particular, sensors which can monitor the local carbon dioxide levels and inflammatory response in loaded skin sites could provide the potential to identify early compromise of tissues prior to gross damage. The modification of existing technologies such as long-term pressure and shear monitoring could also provide a means by which patient posture and mobility can be tracked over prolonged periods. This could inform patients, carers and clinicians of behaviors which will predispose individuals to increased risk of PUs, DFUs. The creation of algorithms which can format and process the large data generated from such sensors could also lead to improved translation of these technologies, with the potential for machine learning to facilitate this process. There is also a significant opportunity to use the bioengineering approaches to optimise the design of medical devices, including their material combinations, in contact with the skin. Future goals related to both PU and DFU prevention, which could be achieved in the research and clinical setting, can be summarised as:

- Establishment of objective risk assessment tools for PU and DFU applications, which are reliable and robust.
- Development of an integrated system to monitor conditions at the loaded body-support interface, including pressure, shear and microclimate (Fig. 2).
- Use of novel materials and advanced support systems to create a ‘closed loop system’ for skin protection.
- Prediction of the interface/interstitial conditions which may lead to tissue breakdown.
- Validation of computational models, which can provide clinical translation for the prevention and management of chronic wounds.

## Abbreviations

PU: pressure ulcer
DFU: diabetic foot ulcer
MRI: magnetic resonance imaging
CT: computer tomography
ECM: extracellular matrix
DTI: deep tissue injury
SCI: spinal cord injury
IAD: incontinence associated dermatitis
ICU: intensive care unit
ADL: activities of daily living
FEA: finite element analysis

## References

[1] Briggaman RA, Wheeler CE (1975). The epidermal–dermal junction. J Investig Dermatol.

[2] Pasparakis M, Haase I, Nestle FO (2014). Mechanisms regulating skin immunity and inflammation. Nat Rev Immunol.

[3] National Pressure Ulcer Advisory Panel, European Pressure Ulcer Advisory Panel, Alliance PPPI (2014). Prevention and treatment of pressure ulcers: quick reference guide.

[4] Vanderwee K, Clark M, Dealey C, Gunningberg L, Defloor T (2007). Pressure ulcer prevalence in Europe: a pilot study. J Eval Clin Pract.

[5] Khanolkar MP, Bain SC, Stephens JW (2008). The diabetic foot. QJM Int J Med.

[6] Boyko EJ, Ahroni JH, Smith DG, Davignon D (1996). Increased mortality associated with diabetic foot ulcer. Diabet Med.

[7] Krentz AJ, Acheson P, Basu A, Kilvert A, Wright AD, Nattrass M (1997). Morbidity and mortality associated with diabetic foot disease: a 12-month prospective survey of hospital admissions in a single UK centre. Foot.

[8] Ahmad N, Thomas GN, Gill P, Torella F (2016). The prevalence of major lower limb amputation in the diabetic and non-diabetic population of England 2003–2013. Diab Vasc Dis Res.

[9] Baharestani MM, Ratcliff CR (2007). Pressure ulcers in neonates and children: an NPUAP white paper. Adv Skin Wound Care.

[10] Smit I, Harrison L, Letzkus L, Quatrara B (2016). What factors are associated with the development of pressure ulcers in a medical intensive care unit?. Dimens Crit Care Nurs.

[11] Lala D, Dumont FS, Leblond J, Houghton PE, Noreau L (2014). Impact of pressure ulcers on individuals living with a spinal cord injury. Arch Phys Med Rehabil.

[12] Guest JF, Ayoub N, McIlwraith T, Uchegbu I, Gerrish A, Weidlich D, Vowden K, Vowden P (2015). Health economic burden that wounds impose on the national health service in the UK. BMJ Open.

[13] Kasuya A, Sakabe JI, Tokura Y (2014). Potential application of in vivo imaging of impaired lymphatic duct to evaluate the severity of pressure ulcer in mouse model. Sci Rep.

[14] Gray RJ, Voegeli D, Bader DL (2016). Features of lymphatic dysfunction in compressed skin tissues—implications in pressure ulcer aetiology. J Tissue Viability.

[15] Gray RJ, Worsley PR, Voegeli D, Bader DL (2016). Monitoring contractile dermal lymphatic activity following uniaxial mechanical loading. Med Eng Phys.

[16] Jiang LP, Tu Q, Wang Y, Zhang E (2011). Ischemia-reperfusion injury-induced histological changes affecting early stage pressure ulcer development in a rat model. Ostomy Wound Manage.

[17] Peirce SM, Skalak TC, Rodeheaver GT (2000). Ischemia-reperfusion injury in chronic pressure ulcer formation: a skin model in the rat. Wound Repair Regen.

[18] Bouten C, Oomens C, Baaijens F, Bader D (2003). The etiology of pressure ulcers: skin deep or muscle bound?. Arch Phys Med Rehabil.

[19] Linder-Ganz E, Engelberg S, Scheinowitz M, Gefen A (2006). Pressure–time cell death threshold for albino rat skeletal muscles as related to pressure sore biomechanics. J Biomech.

[20] Stekelenburg A, Strijkers GJ, Parusel H, Bader DL, Nicolay K, Oomens CW (2007). Role of ischemia and deformation in the onset of compression-induced deep tissue injury: MRI-based studies in a rat model. J Appl Physiol.

[21] Noor S, Zubair M, Ahmad J (2015). Diabetic foot ulcer—a review on pathophysiology, classification and microbial etiology. Diabetes Metab Syndr Clin Res Rev.

[22] Gefen A (2009). Reswick and Rogers pressure-time curve for pressure ulcer risk. Part 2. Nurs Stand.

[23] Reswick JB, Rogers JE. Experience at Rancho Los Amigos hospital with devices and techniques to prevent pressure sores. In: Kenedi RM, Cowden JM, editors. Bed sore biomechanics: proceedings of a seminar on tissue viability and clinical applications organised in association with the department of biomedical engineering, the institute of orthopaedics (University of London), royal national orthopaedic hospital, Stanmore, London, and held at the University of Strathclyde, Glasgow, in August, 1975. London: Macmillan Education UK; 1976. p. 301–10.

[24] Gefen A, van Nierop B, Bader D, Oomens C (2008). Strain-time cell-death threshold for skeletal muscle in a tissue-engineered model system for deep tissue injury. J Biomech.

[25] Coleman S, Nixon J, Keen J, Wilson L, McGinnis E, Dealey C, Stubbs N, Farrin A, Dowding D, Schols JM (2014). A new pressure ulcer conceptual framework. J Adv Nurs.

[26] Linder-Ganz E, Shabshin N, Itzchak Y, Gefen A (2007). Assessment of mechanical conditions in sub-dermal tissues during sitting: a combined experimental-MRI and finite element approach. J Biomech.

[27] Sonenblum SE, Sprigle SH, Martin JS (2016). Everyday sitting behavior of full-time wheelchair users. J Rehabil Res Dev.

[28] Walia GS, Wong AL, Lo AY, Mackert GA, Carl HM, Pedreira RA, Bello R, Aquino CS, Padula WV, Sacks JM (2016). Efficacy of monitoring devices in support of prevention of pressure injuries: systematic review and meta-analysis. Adv Skin Wound Care.

[29] Siddiqui A, Behrendt R, Lafluer M, Craft S (2013). A continuous bedside pressure mapping system for prevention of pressure ulcer development in the medical ICU: a retrospective analysis. Wounds.

[30] Behrendt R, Ghaznavi AM, Mahan M, Craft S, Siddiqui A (2014). Continuous bedside pressure mapping and rates of hospital-associated pressure ulcers in a medical intensive care unit. Am J Crit Care.

[31] Gefen A (2007). Pressure-sensing devices for assessment of soft tissue loading under bony prominences: technological concepts and clinical utilization. Wounds.

[32] Gunningberg L, Carli C (2016). Reduced pressure for fewer pressure ulcers: can real-time feedback of interface pressure optimise repositioning in bed?. Int Wound J.

[33] Worsley PR, Rebolledo D, Webb S, Caggiari S, Bader DL (2017). Monitoring the biomechanical and physiological effects of postural changes during leisure chair sitting. J Tissue Viability.

[34] Kim JH, Wang XL, Ho CH, Bogie KM (2012). Physiological measurements of tissue health; implications for clinical practice. Int Wound J.

[35] Woodhouse M, Worsley PR, Voegeli D, Schoonhoven L, Bader DL (2015). The physiological response of soft tissue to periodic repositioning as a strategy for pressure ulcer prevention. Clin Biomech.

[36] Worsley PR, Parsons B, Bader DL (2016). An evaluation of fluid immersion therapy for the prevention of pressure ulcers. Clin Biomech.

[37] Hemmes B, Poeze M, Brink PR (2010). Reduced tissue-interface pressure and increased comfort on a newly developed soft-layered long spineboard. J Trauma.

[38] Stockton L, Gebhardt KS, Clark M (2009). Seating and pressure ulcers: clinical practice guideline. J Tissue Viability.

[39] Worsley PR, Prudden G, Gower G, Bader DL (2016). Investigating the effects of strap tension during non-invasive ventilation mask application: a combined biomechanical and biomarker approach. Med Devices (Auckland, NZ).

[40] Ledoux WR, Shofer JB, Cowley MS, Ahroni JH, Cohen V, Boyko EJ (2013). Diabetic foot ulcer incidence in relation to plantar pressure magnitude and measurement location. J Diabetes Complicat.

[41] Laszczak P, McGrath M, Tang J, Gao J, Jiang L, Bader DL, Moser D, Zahedi S (2016). A pressure and shear sensor system for stress measurement at lower limb residuum/socket interface. Med Eng Phys.

[42] Laszczak P, Jiang L, Bader DL, Moser D, Zahedi S (2015). Development and validation of a 3D-printed interfacial stress sensor for prosthetic applications. Med Eng Phys.

[43] Gerhardt LC, Strassle V, Lenz A, Spencer ND, Derler S (2008). Influence of epidermal hydration on the friction of human skin against textiles. J R Soc Interface.

[44] Derler S, Schrade U, Gerhardt LC (2007). Tribology of human skin and mechanical skin equivalents in contact with textiles. Wear.

[45] Figliola RS (2003). A proposed method for quantifying low-air-loss mattress performance by moisture transport. Ostomy Wound Manage.

[46] Patel S, Knapp CF, Donofrio JC, Salcido R (1999). Temperature effects on surface pressure-induced changes in rat skin perfusion: implications in pressure ulcer development. J Rehabil Res Dev.

[47] Du Bois E (1921). The basal metabolism in fever. J Am Med Assoc.

[48] Sandoval-Palomares JDJ, Yáñez-Mendiola J, Gómez-Espinosa A, López-Vela JM (2016). Portable system for monitoring the microclimate in the footwear-foot interface. Sensors (Basel, Switzerland).

[49] Han Y, Liu F, Dowd G, Zhe J (2015). A thermal management device for a lower-limb prosthesis. Appl Therm Eng.

[50] Lo WT, Yick KL, Ng SP, Yip J (2014). New methods for evaluating physical and thermal comfort properties of orthotic materials used in insoles for patients with diabetes. J Rehabil Res Dev.

[51] Yusuf S, Okuwa M, Shigeta Y, Dai M, Iuchi T, Rahman S, Usman A, Kasim S, Sugama J, Nakatani T, Sanada H (2015). Microclimate and development of pressure ulcers and superficial skin changes. Int Wound J.

[52] Sae-Sia W, Wipke-Tevis DD, Williams DA (2005). Elevated sacral skin temperature (T(s)): a risk factor for pressure ulcer development in hospitalized neurologically impaired Thai patients. Appl Nurs Res.

[53] Gefen A (2011). How do microclimate factors affect the risk for superficial pressure ulcers: a mathematical modeling study. J Tissue Viability.

[54] Orsted H, Ohura T, Harding K. Pressure ulcer prevention. Pressure, shear, friction and microclimate in context. International Review; 2010. pp. 1–25.

[55] Atherton DJ (2004). A review of the pathophysiology, prevention and treatment of irritant diaper dermatitis. Curr Med Res Opin.

[56] Reger SI, Ranganathan VK, Sahgal V (2007). Support surface interface pressure, microenvironment, and the prevalence of pressure ulcers: an analysis of the literature. Ostomy Wound Manage.

[57] Zhong W, Xing MM, Pan N, Maibach HI (2006). Textiles and human skin, microclimate, cutaneous reactions: an overview. Cutan Ocul Toxicol.

[58] Bogie KM, Wang XL, Fei B, Sun J (2008). New technique for real-time interface pressure analysis: getting more out of large image data sets. J Rehabil Res Dev.

[59] Reenalda J, Jannink M, Nederhand M, Ijzerman M (2009). Clinical use of interface pressure to predict pressure ulcer development: a systematic review. Assist Technol.

[60] Bennetts CJ, Owings TM, Erdemir A, Botek G, Cavanagh PR (2013). Clustering and classification of regional peak plantar pressures of diabetic feet. J Biomech.

[61] Bates-Jensen BM, McCreath HE, Pongquan V, Apeles NCR (2008). Subepidermal moisture differentiates erythema and stage I pressure ulcers in nursing home residents. Wound Repair Regen.

[62] Worsley PR, Voegeli D (2013). Back to basics: biophysical methods in tissue viability research. J Wound Care.

[63] Kottner J, Dobos G, Andruck A, Trojahn C, Apelt J, Wehrmeyer H, Richter C, Blume-Peytavi U (2015). Skin response to sustained loading: a clinical explorative study. J Tissue Viability.

[64] Schario M, Tomova-Simitchieva T, Lichterfeld A, Herfert H, Dobos G, Lahmann N, Blume-Peytavi U, Kottner J (2017). Effects of two different fabrics on skin barrier function under real pressure conditions. J Tissue Viability.

[65] Filon FL, D’Agostin F, Crosera M, Adami G, Bovenzi M, Maina G (2009). In vitro absorption of metal powders through intact and damaged human skin. Toxicol In Vitro.

[66] Andersen ES, Karlsmark T (2008). Evaluation of four non-invasive methods for examination and characterization of pressure ulcers. Skin Res Technol.

[67] Scheel-Sailer A, Frotzler A, Mueller G, Annaheim S, Rossi RM, Derler S (2017). Biophysical skin properties of grade 1 pressure ulcers and unaffected skin in spinal cord injured and able-bodied persons in the unloaded sacral region. J Tissue Viability.

[68] Scheel-Sailer A, Frotzler A, Mueller G, Annaheim S, Rossi RM, Derler S (2015). Challenges to measure hydration, redness, elasticity and perfusion in the unloaded sacral region of healthy persons after supine position. J Tissue Viability.

[69] Chilcott RP, Farrar R (2000). Biophysical measurements of human forearm skin in vivo: effects of site, gender, chirality and time. Skin Res Technol.

[70] Chai CY, Bader DL (2013). The physiological response of skin tissues to alternating support pressures in able-bodied subjects. J Mech Behav Biomed Mater.

[71] Goossens RH, Rithalia SV (2008). Physiological response of the heel tissue on pressure relief between three alternating pressure air mattresses. J Tissue Viability.

[72] Wang Z, Hasan R, Firwana B, Elraiyah T, Tsapas A, Prokop L, Mills JL, Murad MH (2016). A systematic review and meta-analysis of tests to predict wound healing in diabetic foot. J Vasc Surg.

[73] Bogie KM, Nuseibeh I, Bader DL (1995). Early progressive changes in tissue viability in the seated spinal cord injured subject. Paraplegia.

[74] Mirtaheri P, Gjovaag T, Worsley PR, Bader DL (2015). A review of the role of the partial pressure of carbon dioxide in mechanically loaded tissues: the canary in the cage singing in tune with the pressure ulcer mantra. Ann Biomed Eng.

[75] Park HS, Yun HM, Jung IM, Lee T (2016). Role of laser doppler for the evaluation of pedal microcirculatory function in diabetic neuropathy patients. Microcirculation.

[76] Lindgren M, Malmqvist LA, Sjoberg F, Ek AC (2006). Altered skin blood perfusion in areas with non blanchable erythema: an explorative study. Int Wound J.

[77] Nixon J, Smye S, Scott J, Bond S (1999). The diagnosis of early pressure sores: report of the pilot study. J Tissue Viability.

[78] Forsythe RO, Hinchliffe RJ (2016). Assessment of foot perfusion in patients with a diabetic foot ulcer. Diabetes Metab Res Rev.

[79] Manorama AA, Baek S, Vorro J, Sikorskii A, Bush TR (2010). Blood perfusion and transcutaneous oxygen level characterizations in human skin with changes in normal and shear loads—implications for pressure ulcer formation. Clin Biomech.

[80] Wang X-Q, Mill J, Kravchuk O, Kimble RM (2010). Ultrasound assessed thickness of burn scars in association with laser Doppler imaging determined depth of burns in paediatric patients. Burns.

[81] Petersen LJ (2013). Direct comparison of laser Doppler flowmetry and laser Doppler imaging for assessment of experimentally-induced inflammation in human skin. Inflamm Res.

[82] Knight S, Taylor R, Polliak A, Bader DL (2001). Establishing predictive indicators for the status of loaded soft tissues. J Appl Physiol.

[83] de Wert LA, Bader DL, Oomens CW, Schoonhoven L, Poeze M, Bouvy ND (2015). A new method to evaluate the effects of shear on the skin. Wound Repair Regen.

[84] Hatanaka N, Yamamoto Y, Ichihara K, Mastuo S, Nakamura Y, Watanabe M, Iwatani Y (2008). A new predictive indicator for development of pressure ulcers in bedridden patients based on common laboratory tests results. J Clin Pathol.

[85] Soetens J, Worsley PR, Oomens C, Bader DL. Early detection of skin damage using biomarkers. In: European pressure ulcer advisory panel conference. Belfast; 2017.

[86] Bader DL, Bouten C, Colin D, Oomens C (2005). Pressure ulcer research: current and future perspectives.

[87] Herniman J, Langley GJ, Greenhill R, Worsley PR, Bader DL, Jenkins T. The analysis of sweat biomarkers in mechanically-loaded tissues using SFC-MS. In: American society for mass spectrometry annual conference (ASMS). USA; 2015.

[88] Hemmes B, de Wert LA, Brink PRG, Oomens CWJ, Bader DL, Poeze M (2017). Cytokine IL1alpha and lactate as markers for tissue damage in spineboard immobilisation. A prospective, randomised open-label crossover trial. J Mech Behav Biomed Mater.

[89] Loerakker S, Huisman ES, Seelen HA, Glatz JF, Baaijens FP, Oomens CW, Bader DL (2012). Plasma variations of biomarkers for muscle damage in male nondisabled and spinal cord injured subjects. J Rehabil Res Dev.

[90] Bosboom EM, Bouten CV, Oomens CW, van Straaten HW, Baaijens FP, Kuipers H (2001). Quantification and localisation of damage in rat muscles after controlled loading; a new approach to study the aetiology of pressure sores. Med Eng Phys.

[91] Stekelenburg A, Oomens CW, Strijkers GJ, Nicolay K, Bader DL (1985). Compression-induced deep tissue injury examined with magnetic resonance imaging and histology. J Appl Physiol.

[92] Loerakker S, Stekelenburg A, Strijkers GJ, Rijpkema JJ, Baaijens FP, Bader DL, Nicolay K, Oomens CW (2010). Temporal effects of mechanical loading on deformation-induced damage in skeletal muscle tissue. Ann Biomed Eng.

[93] Oomens CW, Zenhorst W, Broek M, Hemmes B, Poeze M, Brink PR, Bader DL (2013). A numerical study to analyse the risk for pressure ulcer development on a spine board. Clin Biomech (Bristol, Avon).

[94] Portnoy S, Yizhar Z, Shabshin N, Itzchak Y, Kristal A, Dotan-Marom Y, Siev-Ner I, Gefen A (2008). Internal mechanical conditions in the soft tissues of a residual limb of a trans-tibial amputee. J Biomech.

[95] Akins JS, Vallely JJ, Karg PE, Kopplin K, Gefen A, Poojary-Mazzotta P, Brienza DM (2016). Feasibility of freehand ultrasound to measure anatomical features associated with deep tissue injury risk. Med Eng Phys.

[96] Stekelenburg A, Oomens CWJ, Strijkers GJ, Nicolay K, Bader DL (2006). Compression-induced deep tissue injury examined with magnetic resonance imaging and histology. J Appl Physiol.

[97] Loerakker S, Manders E, Strijkers GJ, Nicolay K, Baaijens F, Bader D, Oomens C (2011). The effects of deformation, ischemia, and reperfusion on the development of muscle damage during prolonged loading. J Appl Physiol.

[98] Loerakker S, Oomens C, Manders E, Schakel T, Bader D, Baaijens F, Nicolay K, Strijkers GJ (2011). Ischemia-reperfusion injury in rat skeletal muscle assessed with T2-weighted and dynamic contrast-enhanced MRI. Med Reson Med.

[99] Loerakker S, Solis L, Bader D, Baaijens F, Muchahwar V, Oomens C (2012). How does muscle stiffness affect the internal deformations within the soft tissue layers of the buttocks under constant loading?. Comput Methods Biomech Biomed Eng.

[100] Dickinson AS, Steer JW, Worsley PR (2017). Finite element analysis of the amputated lower limb: a systematic review and recommendations. Med Eng Phys.

[101] Petre M, Erdemir A, Cavanagh PR (2008). An MRI-compatible foot-loading device for assessment of internal tissue deformation. J Biomech.

[102] Makhsous M, Lin F, Cichowski A, Cheng I, Fasanati C, Grant T, Hendrix RW (2011). Use of MRI images to measure tissue thickness over the ischial tuberosity at different hip flexion. Clin Anat.

[103] Call E, Hetzel T, McLean C, Burton JN, Oberg C (2017). Off loading wheelchair cushion provides best case reduction in tissue deformation as indicated by MRI. J Tissue Viability.

[104] Sonenblum SE, Sprigle SH, Cathcart JM, Winder RJ (2015). 3D anatomy and deformation of the seated buttocks. J Tissue Viability.

[105] Brienza D, Vallely J, Karg P, Akins J, Gefen A (2017). An MRI investigation of the effects of user anatomy and wheelchair cushion type on tissue deformation. J Tissue Viability.

[106] Luboz V, Petrizelli M, Bucki M, Diot B, Vuillerme N, Payan Y (2014). Biomechanical modeling to prevent ischial pressure ulcers. J Biomech.

[107] Bucki M, Luboz V, Perrier A, Champion E, Diot B, Vuillerme N, Payan Y (2016). Clinical workflow for personalized foot pressure ulcer prevention. Med Eng Phys.

[108] Faustini MC, Neptune RR, Crawford RH (2006). The quasi-static response of compliant prosthetic sockets for transtibial amputees using finite element methods. Med Eng Phys.

[109] Barwick A, Tessier J, Mirow J, de Jonge XJ, Chuter V (2017). Computed tomography derived bone density measurement in the diabetic foot. J Foot Ankle Res.

[110] Kalra MK, Maher MM, Toth TL, Hamberg LM, Blake MA, Shepard J-A, Saini S (2004). Strategies for CT radiation dose optimization. Radiology.

[111] Quintavalle PR, Lyder CH, Mertz PJ, Phillips-Jones C, Dyson M (2006). Use of high-resolution, high-frequency diagnostic ultrasound to investigate the pathogenesis of pressure ulcer development. Adv Skin Wound Care.

[112] Telfer S, Woodburn J, Turner DE (2014). Measurement of functional heel pad behaviour in-shoe during gait using orthotic embedded ultrasonography. Gait Posture.

[113] Aoi N, Yoshimura K, Kadono T, Nakagami G, Iizuka S, Higashino T, Araki J, Koshima I, Sanada H (2009). Ultrasound assessment of deep tissue injury in pressure ulcers: possible prediction of pressure ulcer progression. Plast Reconstr Surg.

[114] Swaine JM, Moe A, Breidahl W, Bader DL, Oomens CWJ, Lester L, O’Loughlin E, Santamaria N, Stacey MC (2017). Adaptation of a MR imaging protocol into a real-time clinical biometric ultrasound protocol for persons with spinal cord injury at risk for deep tissue injury: a reliability study. J Tissue Viability.

[115] Muthupillai R, Ehman RL (1996). Magnetic resonance elastography. Nat Med.

[116] Ophir J, Cespedes I, Ponnekanti H, Yazdi Y, Li X (1991). Elastography: a quantitative method for imaging the elasticity of biological tissues. Ultrason Imaging.

[117] Cheung YY, Doyley M, Miller TB, Kennedy F, Lynch F, Wrobel JS, Paulson K, Weaver J (2006). Magnetic resonance elastography of the plantar fat pads: preliminary study in diabetic patients and asymptomatic volunteers. J Comput Assist Tomogr.

[118] Nelissen JL, de Graaf L, Traa WA, Schreurs TJ, Moerman KM, Nederveen AJ, Sinkus R, Oomens CW, Nicolay K, Strijkers GJ (2017). A MRI-compatible combined mechanical loading and MR elastography setup to study deformation-induced skeletal muscle damage in rats. PLoS ONE.

[119] Deprez J-F, Brusseau E, Fromageau J, Cloutier G, Basset O (2011). On the potential of ultrasound elastography for pressure ulcer early detection. Med Phys.

[120] Miller GE, Seale J (1981). Lymphatic clearance during compressive loading. Lymphology.

[121] Miller GE, Seale JL (1987). The recovery of terminal lymph flow following occlusion. J Biomech Eng.

[122] Moerman KM, van Vijven M, Solis LR, van Haaften EE, Loenen ACY, Mushahwar VK, Oomens CWJ (2017). On the importance of 3D, geometrically accurate, and subject-specific finite element analysis for evaluation of in vivo soft tissue loads. Comput Methods Biomech Biomed Eng.

[123] Nakagami G, Sanada H, Iizaka S, Kadono T, Higashino T, Koyanagi H, Haga N (2010). Predicting delayed pressure ulcer healing using thermography: a prospective cohort study. J Wound Care.

[124] Lee VSP, Gross P, Spence WD, Solomonidis SE, Paul JP (1998). Two dimensional finite element model of a transverse section of the trans-femoral amputee’s stump. Comput Methods Biomech Biomed Eng.

[125] Oomens CWJ, Bressers OFJT, Bosboom EMH, Bouten CVC, Bader DL (2003). Can loaded interface characteristics influence strain distributions in muscle adjacent to bony prominences?. Comput Methods Biomech Biomed Eng.

[126] Bosboom EMH, Hesselink MKC, Oomens CWJ, Bouten CVC, Drost MR, Baaijens FPT (2001). Passive transverse mechanical properties of skeletal muscle under in vivo compression. J Biomech.

[127] Palevski A, Glaich I, Portnoy S, Linder-Ganz E, Gefen A (2006). Stress relaxation of porcine gluteus muscle subjected to sudden transverse deformation as related to pressure sore modeling. J Biomech Eng.

[128] Groves RB, Coulman SA, Birchall JC, Evans SL (2013). An anisotropic, hyperelastic model for skin: experimental measurements, finite element modelling and identification of parameters for human and murine skin. J Mech Behav Biomed Mater.

[129] Hendriks FM, Brokken D, Van Eemeren JTWM, Oomens CWJ, Baaijens FPT, Horsten JBAM (2003). A numerical-experimental method to characterize the non-linear mechanical behaviour of human skin. Skin Res Technol.

[130] Alkhouli N, Mansfield J, Green E, Bell J, Knight B, Liversedge N, Tham JC, Welbourn R, Shore AC, Kos K, Winlove CP (2013). The mechanical properties of human adipose tissues and their relationships to the structure and composition of the extracellular matrix. Am J Physiol Endocrinol Metab.

[131] Freutel M, Schmidt H, Dürselen L, Ignatius A, Galbusera F (2014). Finite element modeling of soft tissues: material models, tissue interaction and challenges. Clin Biomech.

[132] Then C, Menger J, Vogl TJ, Hubner F, Silber G (2009). Mechanical gluteal soft tissue material parameter validation under complex tissue loading. Technol Health Care.

[133] Loerakker S, Manders E, Strijkers GJ, Nicolay K, Baaijens FP, Bader DL, Oomens CW (1985). The effects of deformation, ischemia, and reperfusion on the development of muscle damage during prolonged loading. J Appl Physiol.

[134] Oomens CWJ, Loerakker S, Bader DL (2010). The importance of internal strain as opposed to interface pressure in the prevention of pressure related deep tissue injury. J Tissue Viability.

[135] Levy A, Kopplin K, Gefen A (2014). An air-cell-based cushion for pressure ulcer protection remarkably reduces tissue stresses in the seated buttocks with respect to foams: finite element studies. J Tissue Viability.

[136] Lee W, Won BH, Cho SW (2017). Finite element modeling for predicting the contact pressure between a foam mattress and the human body in a supine position. Comput Methods Biomech Biomed Eng.

[137] Oomens CWJ, Broek M, Hemmes B, Bader DL (2016). How does lateral tilting affect the internal strains in the sacral region of bed ridden patients?—a contribution to pressure ulcer prevention. Clin Biomech.

[138] Leung IPH, Fleming L, Walton K, Barrans S, Ousey K (2017). Development of a model to demonstrate the effects of friction and pressure on skin in relation to pressure ulcer formation. Wear.

[139] Shaked E, Gefen A (2013). Modeling the effects of moisture-related skin-support friction on the risk for superficial pressure ulcers during patient repositioning in bed. Front Bioeng Biotechnol.

[140] Sopher R, Gefen A (2011). Effects of skin wrinkles, age and wetness on mechanical loads in the stratum corneum as related to skin lesions. Med Biol Eng Comput.

[141] Levy A, Frank MBO, Gefen A (2015). The biomechanical efficacy of dressings in preventing heel ulcers. J Tissue Viability.

[142] Telfer S, Erdemir A, Woodburn J, Cavanagh PR (2014). What has finite element analysis taught us about diabetic foot disease and its management? A systematic review. PLOS ONE.

[143] Levy A, Kopplin K, Gefen A (2017). Device-related pressure ulcers from a biomechanical perspective. J Tissue Viability.

[144] Koppenol DC, Vermolen FJ, Niessen FB, van Zuijlen PPM, Vuik K (2017). A mathematical model for the simulation of the formation and the subsequent regression of hypertrophic scar tissue after dermal wounding. Biomech Model Mechanobiol.

[145] Luboz V, Bailet M, Boichon Grivot C, Rochette M, Diot B, Bucki M, Payan Y (2017). Personalized modeling for real-time pressure ulcer prevention in sitting posture. J Tissue Viability.

[146] Vogl TJ, Then C, Naguib NNN, Nour-Eldin NEA, Larson M, Zangos S, Silber G (2010). Mechanical soft tissue property validation in tissue engineering using magnetic resonance imaging. Acad Radiol.

